# 3,5-Bis(4-fluoro­phen­yl)-1-phenyl-4,5-dihydro-1*H*-pyrazole

**DOI:** 10.1107/S1600536810026036

**Published:** 2010-07-07

**Authors:** Jerry P. Jasinski, Curtis J. Guild, S. Samshuddin, B. Narayana, H. S. Yathirajan

**Affiliations:** a Department of Chemistry, Keene State College, 229 Main Street, Keene, NH 03435-2001, USA; b Department of Studies in Chemistry, Mangalore University, Mangalagangotri 574 199, India; c Department of Studies in Chemistry, University of Mysore, Manasagangotri, Mysore 570 006, India

## Abstract

In the title compound, C_21_H_16_F_2_N_2_, the dihedral angle between the fluoro­phenyl groups is 66.34 (8)°, and the dihedral angle between the envelope-configured pyrazole group (N/N/C/C/C) and the benzene ring is 11.50 (9)°. The dihedral angles between the benzene and the two fluoro-substituted phenyl groups are 77.7 (6) and 16.7 (5)°. Weak C—H⋯π interactions contribute to the stability of the crystal structure.

## Related literature

For background to the chemistry and biological activity of pyrazolines, see: Amir *et al.* (2008 ▶); Bhaskarreddy *et al.* (1997 ▶); Fustero *et al.* (2009 ▶); Hes *et al.* (1978 ▶); Klimova *et al.* (1999 ▶); Regaila *et al.* (1979 ▶); Sarojini *et al.* (2010 ▶); Wiley *et al.* (1958 ▶); Spek (2009 ▶). For related structures, see: Butcher *et al.* (2007 ▶); Fun, Quah *et al.* (2009 ▶); Fun, Yeap *et al.* (2009 ▶); Fun *et al.* (2010 ▶); Guo *et al.* (2006 ▶, 2007 ▶); Li (2007*a* ▶,*b* ▶); Loh *et al.* (2010 ▶); Yathirajan *et al.* (2007*a* ▶,*b* ▶).
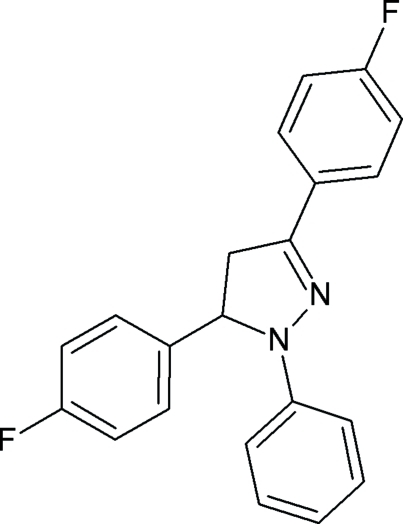


## Experimental

### 

#### Crystal data

C_21_H_16_F_2_N_2_
*M* *_r_* = 334.36
Monoclinic, 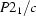

*a* = 12.2880 (3) Å
*b* = 13.1678 (3) Å
*c* = 11.3245 (3) Å
β = 112.661 (3)°
*V* = 1690.91 (7) Å^3^
*Z* = 4
Cu *K*α radiation
μ = 0.77 mm^−1^
*T* = 100 K
0.28 × 0.24 × 0.23 mm

#### Data collection

Oxford Diffraction Xcalibur diffractometer with a Ruby (Gemini Cu) detector
Absorption correction: multi-scan (*CrysAlis RED*; Oxford Diffraction, 2007 ▶) *T*_min_ = 0.774, *T*_max_ = 1.000
7737 measured reflections
3541 independent reflections
2740 reflections with *I* > 2σ(*I*)
*R*_int_ = 0.016

#### Refinement

*R*[*F* ^2^ > 2σ(*F* ^2^)] = 0.039
*wR*(*F* ^2^) = 0.116
*S* = 1.05
3541 reflections
226 parameters
H-atom parameters constrained
Δρ_max_ = 0.15 e Å^−3^
Δρ_min_ = −0.16 e Å^−3^

### 

Data collection: *CrysAlis PRO* (Oxford Diffraction, 2007 ▶); cell refinement: *CrysAlis RED* (Oxford Diffraction, 2007 ▶); data reduction: *CrysAlis RED*; program(s) used to solve structure: *SHELXS97* (Sheldrick, 2008 ▶); program(s) used to refine structure: *SHELXL97* (Sheldrick, 2008 ▶); molecular graphics: *SHELXTL* (Sheldrick, 2008 ▶); software used to prepare material for publication: *SHELXTL* and *PLATON* (Spek, 2009 ▶).

## Supplementary Material

Crystal structure: contains datablocks global, I. DOI: 10.1107/S1600536810026036/tk2686sup1.cif

Structure factors: contains datablocks I. DOI: 10.1107/S1600536810026036/tk2686Isup2.hkl

Additional supplementary materials:  crystallographic information; 3D view; checkCIF report

## Figures and Tables

**Table 1:** *Y*—*X*⋯*Cg* π ring inter­actions (Å, °) *Cg*4 is the centroid of ring C16–C21 and *Cg*2 is the centroid of the ring C1–C6.

*X*—*H*⋯*CgX*	*X*⋯*Cg*	H⋯*Cg*	*X*⋯Perp
C9—H9⋯*Cg*4^i^	3.6677 (16)	2.82	2.76
C12—H12⋯*Cg*2^ii^	3.6061 (18)	2.88	−2.79

Symmetry codes: (i) 1 − *x*, 1 − *y*, 1 − *z*; (ii) −*x*, −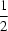 + *y*, 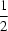 − *z*.

**Fig. 1.**
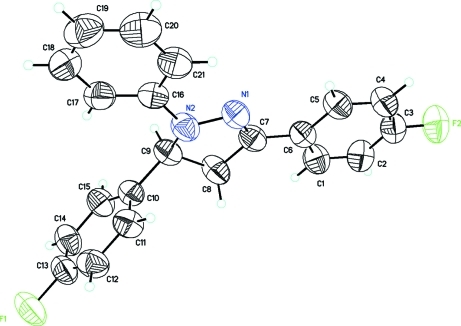
Molecular structure of (I), with 50% probability displacement ellipsoids.

**Fig. 2.**
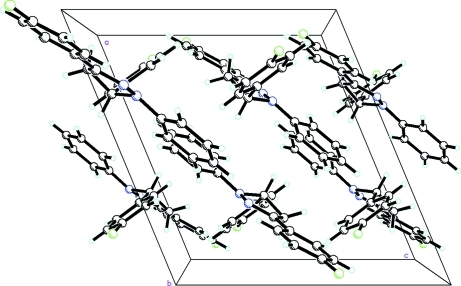
Packing diagram for (I), viewed down the c axis.

## supplementary crystallographic information

### Comment

Pyrazolines are well known as important nitrogen-containing five-membered heterocyclic compounds and various methods have been worked out for their synthesis (Fustero *et al.*, 2009). The pyrazoline function is quite stable and has inspired chemists to utilize this stable fragment in bioactive moieties to synthesize new compounds possessing biological activities, and the presence of fluorine in the molecules at strategic positions alters their activity. Several pyrazoline derivatives have been found to possess considerable biological activities, which stimulated research activity in this field. In particular, they are used as antitumor, antibacterial, antifungal, antiviral, anti-parasitic, anti-tubercular and insecticidal agents (Hes *et al.*, 1978; Amir *et al.*, 2008). Some of these compounds have also anti-inflammatory, anti-diabetic, anaesthetic and analgesic properties (Sarojini *et al.*, 2010; Regaila *et al.*, 1979). Several 1,3,5-triaryl-2 -pyrazolines were also used as scintillation solutes (Wiley *et al.*, 1958). In addition, pyrazolines have played a crucial part in the development of theory in heterocyclic chemistry and also used extensively in organic synthesis (Klimova *et al.*, 1999; Bhaskarreddy *et al.*, 1997).

The crystal structures of some substituted 4,5-dihydro N– phenyl pyrazoles *viz*., 6-chloro-3-[5-(4-fluorophenyl)-1-phenyl-4,5- dihydro-1*H*-pyrazol-3-yl]-2-methyl-4-phenyl quinoline (Loh *et al.*, 2010), 6-chloro-3-[5-(3-methoxy-8-methyl-4- quinolyl)-1-phenyl-4,5-dihydro-1*H*-pyrazol-3-yl]-2-methyl-4-phenyl quinoline (Fun *et al.*, 2009*a*), 6-chloro-2-methyl-4-phenyl- 3-[1-phenyl-5-(2-thienyl)-4,5-dihydro-1*H*-pyrazol-3-yl] quinoline (Fun *et al.*, 2009*b*), 3-(4-fluorophenyl)-1,5-diphenyl-2-pyrazoline (Guo *et al.*, 2006), 3-(4-bromophenyl)-5- (2-chlorophenyl)-1-phenyl-2-pyrazoline, (Guo *et al.*, 2007), 5-(*p*-fluorophenyl)-1,3-diphenyl-2-pyrazoline, 3-(4-bromophenyl)-5 -(4-fluorophenyl)-1-phenyl-4,5-dihydro-1*H*-pyrazole (Li, 2007*a*,b) have been reported. In continuation of our work on pyrazoline derivatives (Fun *et al.*, 2010; Yathirajan *et al.*, 2007*a*,*b*; Butcher *et al.*, 2007) and in view of the importance of these derivatives, the title compound C_21_H_15_N_2_F_2_ (I) was synthesized and its crystal structure is reported here.

The title compound (I) contains two *p*-florophenyl groups and a benzene ring attached to an envelope configured pyrazole ring (Fig. 1). The dihedral angle between the two flourophenyl groups is 66.34 (8)° and the dihedral angle between the pyrazole and benzene rings is 11.50 (9) °. Also, the dihedral angles between the benzene ring and the two fluoro-substituted phenyl groups are 77.7 (6) and 16.7 (5) °, respectively. Two C–H···π interactions (Table 1) contribute to the stability of the crystal structure (Fig. 2).

### Experimental

A mixture of (2*E*)-1,3-bis(4-fluorophenyl)prop-2-en-1-one (2.44 g, 0.01 mol) and phenyl hydrazine (1.08 g, 0.01 mol) in ethanol (20 ml) in the presence of glacial acetic acid (5 ml) was refluxed for 5 h. The reaction mixture was cooled and poured into ice-cold water (50 ml). The precipitate was collected by filtration and purified by recrystallization from ethanol. The single-crystal was grown from toluene by the slow evaporation method. The yield of the compound was 84%; m.pt. 387 K. Analytical data: Found (Calculated): C %: 67.86 (67.99); H %: 4.62 (4.70); N %: 9.29 (9.33).

### Refinement

All of the H atoms were placed in their calculated positions and then refined using the riding model approximation with C—H = 0.93–0.98 Å, and with *U*_iso_(H) = 1.19–1.30*U*_eq_(C).

### Crystal data

**Table:**

C_21_H_16_F_2_N_2_	*F*(000) = 696
*M**_r_* = 334.36	*D*_x_ = 1.313 Mg m^−^^3^
Monoclinic, *P*2_1_/*c*	Cu *K*α radiation, λ = 1.54184 Å
Hall symbol: -P 2ybc	Cell parameters from 3839 reflections
*a* = 12.2880 (3) Å	θ = 4.5–77.2°
*b* = 13.1678 (3) Å	µ = 0.77 mm^−^^1^
*c* = 11.3245 (3) Å	*T* = 100 K
β = 112.661 (3)°	Block, colorless
*V* = 1690.91 (7) Å^3^	0.28 × 0.24 × 0.23 mm
*Z* = 4	

### Data collection

**Table:**

Oxford Diffraction Xcalibur diffractometer with a Ruby (Gemini Cu) detector	3541 independent reflections
Radiation source: fine-focus sealed tube	2740 reflections with *I* > 2σ(*I*)
graphite	*R*_int_ = 0.016
Detector resolution: 10.5081 pixels mm^-1^	θ_max_ = 77.4°, θ_min_ = 5.2°
ω scans	*h* = −11→15
Absorption correction: multi-scan (*CrysAlis RED*; Oxford Diffraction, 2007)	*k* = −16→14
*T*_min_ = 0.774, *T*_max_ = 1.000	*l* = −13→14
7737 measured reflections	

### Refinement

**Table:**

Refinement on *F*^2^	Primary atom site location: structure-invariant direct methods
Least-squares matrix: full	Secondary atom site location: difference Fourier map
*R*[*F*^2^ > 2σ(*F*^2^)] = 0.039	Hydrogen site location: inferred from neighbouring sites
*wR*(*F*^2^) = 0.116	H-atom parameters constrained
*S* = 1.05	*w* = 1/[σ^2^(*F*_o_^2^) + (0.060*P*)^2^ + 0.1618*P*] where *P* = (*F*_o_^2^ + 2*F*_c_^2^)/3
3541 reflections	(Δ/σ)_max_ < 0.001
226 parameters	Δρ_max_ = 0.15 e Å^−^^3^
0 restraints	Δρ_min_ = −0.16 e Å^−^^3^

### Special details

**Table:**

Geometry. All e.s.d.'s (except the e.s.d. in the dihedral angle between two l.s. planes) are estimated using the full covariance matrix. The cell e.s.d.'s are taken into account individually in the estimation of e.s.d.'s in distances, angles and torsion angles; correlations between e.s.d.'s in cell parameters are only used when they are defined by crystal symmetry. An approximate (isotropic) treatment of cell e.s.d.'s is used for estimating e.s.d.'s involving l.s. planes.
Refinement. Refinement of *F*^2^ against ALL reflections. The weighted *R*-factor *wR* and goodness of fit *S* are based on *F*^2^, conventional *R*-factors *R* are based on *F*, with *F* set to zero for negative *F*^2^. The threshold expression of *F*^2^ > σ(*F*^2^) is used only for calculating *R*-factors(gt) *etc*. and is not relevant to the choice of reflections for refinement. *R*-factors based on *F*^2^ are statistically about twice as large as those based on *F*, and *R*- factors based on ALL data will be even larger.

### Fractional atomic coordinates and isotropic or equivalent isotropic displacement parameters (Å^2^)

**Table:**

	*x*	*y*	*z*	*U*_iso_*/*U*_eq_	
F1	0.14722 (10)	0.03973 (7)	0.26845 (11)	0.0891 (3)	
F2	0.03475 (11)	0.92804 (9)	0.61215 (12)	0.0958 (4)	
N1	0.27887 (10)	0.60196 (9)	0.37791 (11)	0.0583 (3)	
N2	0.31295 (11)	0.50566 (9)	0.35601 (11)	0.0594 (3)	
C1	0.11598 (14)	0.67082 (12)	0.57694 (14)	0.0649 (4)	
H1	0.1090	0.6062	0.6064	0.078*	
C2	0.06799 (15)	0.75266 (13)	0.61618 (15)	0.0710 (4)	
H2	0.0288	0.7437	0.6712	0.085*	
C3	0.07946 (15)	0.84700 (12)	0.57224 (15)	0.0682 (4)	
C4	0.13479 (15)	0.86269 (12)	0.48921 (17)	0.0725 (4)	
H4	0.1398	0.9276	0.4593	0.087*	
C5	0.18302 (14)	0.78039 (12)	0.45068 (15)	0.0643 (4)	
H5	0.2215	0.7902	0.3951	0.077*	
C6	0.17452 (12)	0.68283 (10)	0.49427 (12)	0.0549 (3)	
C7	0.22462 (12)	0.59450 (11)	0.45457 (12)	0.0554 (3)	
C8	0.21469 (15)	0.48688 (11)	0.49360 (15)	0.0644 (4)	
H8A	0.1353	0.4608	0.4505	0.077*	
H8B	0.2374	0.4812	0.5854	0.077*	
C9	0.30233 (13)	0.43192 (10)	0.44948 (13)	0.0563 (3)	
H9	0.3785	0.4265	0.5218	0.068*	
C10	0.26207 (11)	0.32735 (10)	0.39674 (12)	0.0515 (3)	
C11	0.18963 (13)	0.31090 (11)	0.26960 (13)	0.0603 (3)	
H11	0.1665	0.3654	0.2132	0.072*	
C12	0.15145 (14)	0.21357 (13)	0.22594 (14)	0.0658 (4)	
H12	0.1039	0.2020	0.1405	0.079*	
C13	0.18527 (14)	0.13539 (11)	0.31124 (15)	0.0632 (4)	
C14	0.25607 (15)	0.14782 (11)	0.43752 (15)	0.0662 (4)	
H14	0.2775	0.0929	0.4934	0.079*	
C15	0.29473 (14)	0.24498 (11)	0.47929 (14)	0.0605 (3)	
H15	0.3437	0.2553	0.5646	0.073*	
C16	0.39681 (12)	0.49799 (11)	0.30110 (13)	0.0567 (3)	
C17	0.46147 (13)	0.40957 (13)	0.31169 (15)	0.0660 (4)	
H17	0.4526	0.3556	0.3602	0.079*	
C18	0.53935 (14)	0.40141 (15)	0.25016 (18)	0.0769 (5)	
H18	0.5818	0.3417	0.2573	0.092*	
C19	0.55439 (16)	0.48058 (17)	0.17879 (18)	0.0853 (5)	
H19	0.6060	0.4745	0.1369	0.102*	
C20	0.49212 (17)	0.56887 (17)	0.17010 (18)	0.0838 (5)	
H20	0.5032	0.6231	0.1232	0.101*	
C21	0.41337 (15)	0.57872 (13)	0.22966 (15)	0.0679 (4)	
H21	0.3716	0.6389	0.2222	0.081*	

### Atomic displacement parameters (Å^2^)

**Table:**

	*U*^11^	*U*^22^	*U*^33^	*U*^12^	*U*^13^	*U*^23^
F1	0.1227 (8)	0.0572 (5)	0.1070 (7)	−0.0245 (5)	0.0660 (7)	−0.0303 (5)
F2	0.1130 (8)	0.0748 (7)	0.1084 (8)	0.0252 (6)	0.0524 (7)	−0.0023 (6)
N1	0.0659 (6)	0.0496 (6)	0.0595 (6)	−0.0020 (5)	0.0243 (5)	0.0031 (5)
N2	0.0710 (7)	0.0487 (6)	0.0649 (6)	−0.0046 (5)	0.0332 (6)	0.0029 (5)
C1	0.0796 (9)	0.0579 (8)	0.0594 (8)	0.0042 (7)	0.0292 (7)	0.0100 (6)
C2	0.0806 (10)	0.0748 (11)	0.0610 (8)	0.0123 (8)	0.0309 (7)	0.0080 (7)
C3	0.0734 (9)	0.0615 (9)	0.0660 (8)	0.0099 (7)	0.0226 (7)	−0.0019 (7)
C4	0.0819 (10)	0.0501 (8)	0.0846 (10)	0.0001 (7)	0.0310 (8)	0.0051 (7)
C5	0.0739 (9)	0.0559 (8)	0.0653 (8)	−0.0043 (7)	0.0294 (7)	0.0037 (6)
C6	0.0608 (7)	0.0511 (7)	0.0482 (6)	−0.0024 (6)	0.0160 (5)	0.0006 (5)
C7	0.0637 (7)	0.0505 (7)	0.0492 (6)	−0.0054 (6)	0.0186 (6)	0.0013 (5)
C8	0.0879 (10)	0.0489 (7)	0.0653 (8)	−0.0086 (7)	0.0392 (8)	−0.0038 (6)
C9	0.0655 (7)	0.0490 (7)	0.0521 (7)	−0.0080 (6)	0.0199 (6)	0.0009 (5)
C10	0.0568 (7)	0.0468 (6)	0.0521 (6)	−0.0041 (5)	0.0224 (5)	−0.0005 (5)
C11	0.0666 (8)	0.0574 (8)	0.0536 (7)	−0.0053 (6)	0.0193 (6)	0.0030 (6)
C12	0.0689 (8)	0.0707 (9)	0.0574 (8)	−0.0129 (7)	0.0238 (7)	−0.0140 (7)
C13	0.0790 (9)	0.0481 (7)	0.0783 (9)	−0.0110 (7)	0.0479 (8)	−0.0148 (7)
C14	0.0893 (10)	0.0472 (7)	0.0713 (9)	0.0007 (7)	0.0409 (8)	0.0036 (6)
C15	0.0748 (8)	0.0518 (7)	0.0532 (7)	−0.0019 (6)	0.0229 (6)	0.0018 (6)
C16	0.0554 (7)	0.0578 (8)	0.0543 (7)	−0.0101 (6)	0.0183 (6)	−0.0038 (6)
C17	0.0608 (7)	0.0643 (9)	0.0730 (9)	−0.0073 (7)	0.0260 (7)	0.0002 (7)
C18	0.0634 (8)	0.0789 (11)	0.0888 (11)	−0.0029 (8)	0.0296 (8)	−0.0106 (9)
C19	0.0762 (10)	0.1065 (15)	0.0854 (11)	−0.0113 (10)	0.0445 (9)	−0.0060 (11)
C20	0.0886 (11)	0.0945 (13)	0.0772 (11)	−0.0122 (10)	0.0420 (9)	0.0117 (9)
C21	0.0735 (9)	0.0678 (9)	0.0649 (8)	−0.0065 (7)	0.0295 (7)	0.0055 (7)

### Geometric parameters (Å, °)

**Table:**

F1—C13	1.3658 (16)	C9—H9	0.9800
F2—C3	1.3551 (19)	C10—C15	1.3865 (19)
N1—C7	1.2859 (19)	C10—C11	1.3873 (19)
N1—N2	1.3875 (17)	C11—C12	1.389 (2)
N2—C16	1.3973 (19)	C11—H11	0.9300
N2—C9	1.4787 (17)	C12—C13	1.363 (2)
C1—C2	1.382 (2)	C12—H12	0.9300
C1—C6	1.392 (2)	C13—C14	1.367 (2)
C1—H1	0.9300	C14—C15	1.383 (2)
C2—C3	1.366 (2)	C14—H14	0.9300
C2—H2	0.9300	C15—H15	0.9300
C3—C4	1.372 (3)	C16—C17	1.388 (2)
C4—C5	1.384 (2)	C16—C21	1.398 (2)
C4—H4	0.9300	C17—C18	1.388 (2)
C5—C6	1.395 (2)	C17—H17	0.9300
C5—H5	0.9300	C18—C19	1.374 (3)
C6—C7	1.465 (2)	C18—H18	0.9300
C7—C8	1.503 (2)	C19—C20	1.374 (3)
C8—C9	1.532 (2)	C19—H19	0.9300
C8—H8A	0.9700	C20—C21	1.382 (3)
C8—H8B	0.9700	C20—H20	0.9300
C9—C10	1.5066 (18)	C21—H21	0.9300
			
C7—N1—N2	108.75 (11)	C15—C10—C11	118.74 (13)
N1—N2—C16	118.08 (11)	C15—C10—C9	118.83 (12)
N1—N2—C9	110.87 (11)	C11—C10—C9	122.38 (12)
C16—N2—C9	123.69 (12)	C10—C11—C12	120.48 (13)
C2—C1—C6	121.53 (15)	C10—C11—H11	119.8
C2—C1—H1	119.2	C12—C11—H11	119.8
C6—C1—H1	119.2	C13—C12—C11	118.43 (13)
C3—C2—C1	118.40 (16)	C13—C12—H12	120.8
C3—C2—H2	120.8	C11—C12—H12	120.8
C1—C2—H2	120.8	C12—C13—F1	118.43 (14)
F2—C3—C2	118.86 (16)	C12—C13—C14	123.25 (13)
F2—C3—C4	118.80 (15)	F1—C13—C14	118.31 (14)
C2—C3—C4	122.34 (15)	C13—C14—C15	117.69 (14)
C3—C4—C5	118.93 (15)	C13—C14—H14	121.2
C3—C4—H4	120.5	C15—C14—H14	121.2
C5—C4—H4	120.5	C14—C15—C10	121.40 (13)
C4—C5—C6	120.67 (15)	C14—C15—H15	119.3
C4—C5—H5	119.7	C10—C15—H15	119.3
C6—C5—H5	119.7	C17—C16—N2	121.18 (13)
C1—C6—C5	118.12 (14)	C17—C16—C21	118.80 (14)
C1—C6—C7	120.21 (13)	N2—C16—C21	119.97 (14)
C5—C6—C7	121.67 (13)	C18—C17—C16	120.26 (16)
N1—C7—C6	122.33 (13)	C18—C17—H17	119.9
N1—C7—C8	113.11 (13)	C16—C17—H17	119.9
C6—C7—C8	124.52 (13)	C19—C18—C17	120.75 (18)
C7—C8—C9	101.68 (12)	C19—C18—H18	119.6
C7—C8—H8A	111.4	C17—C18—H18	119.6
C9—C8—H8A	111.4	C20—C19—C18	119.11 (17)
C7—C8—H8B	111.4	C20—C19—H19	120.4
C9—C8—H8B	111.4	C18—C19—H19	120.4
H8A—C8—H8B	109.3	C19—C20—C21	121.30 (17)
N2—C9—C10	114.99 (11)	C19—C20—H20	119.4
N2—C9—C8	100.96 (11)	C21—C20—H20	119.4
C10—C9—C8	113.34 (11)	C20—C21—C16	119.77 (17)
N2—C9—H9	109.1	C20—C21—H21	120.1
C10—C9—H9	109.1	C16—C21—H21	120.1
C8—C9—H9	109.1		
			
C7—N1—N2—C16	−164.89 (12)	N2—C9—C10—C15	153.43 (13)
C7—N1—N2—C9	−13.70 (16)	C8—C9—C10—C15	−91.13 (16)
C6—C1—C2—C3	−0.2 (2)	N2—C9—C10—C11	−29.19 (19)
C1—C2—C3—F2	−178.70 (14)	C8—C9—C10—C11	86.26 (17)
C1—C2—C3—C4	1.2 (3)	C15—C10—C11—C12	−0.5 (2)
F2—C3—C4—C5	178.47 (14)	C9—C10—C11—C12	−177.88 (13)
C2—C3—C4—C5	−1.4 (3)	C10—C11—C12—C13	1.0 (2)
C3—C4—C5—C6	0.7 (2)	C11—C12—C13—F1	179.67 (13)
C2—C1—C6—C5	−0.5 (2)	C11—C12—C13—C14	−0.8 (2)
C2—C1—C6—C7	179.91 (14)	C12—C13—C14—C15	−0.1 (2)
C4—C5—C6—C1	0.2 (2)	F1—C13—C14—C15	179.47 (14)
C4—C5—C6—C7	179.85 (14)	C13—C14—C15—C10	0.7 (2)
N2—N1—C7—C6	−178.39 (12)	C11—C10—C15—C14	−0.4 (2)
N2—N1—C7—C8	−0.60 (16)	C9—C10—C15—C14	177.09 (14)
C1—C6—C7—N1	179.65 (13)	N1—N2—C16—C17	160.01 (13)
C5—C6—C7—N1	0.0 (2)	C9—N2—C16—C17	12.8 (2)
C1—C6—C7—C8	2.1 (2)	N1—N2—C16—C21	−22.60 (19)
C5—C6—C7—C8	−177.51 (14)	C9—N2—C16—C21	−169.85 (13)
N1—C7—C8—C9	13.51 (16)	N2—C16—C17—C18	176.23 (14)
C6—C7—C8—C9	−168.75 (12)	C21—C16—C17—C18	−1.2 (2)
N1—N2—C9—C10	143.41 (12)	C16—C17—C18—C19	0.5 (2)
C16—N2—C9—C10	−67.31 (17)	C17—C18—C19—C20	0.7 (3)
N1—N2—C9—C8	21.03 (14)	C18—C19—C20—C21	−1.2 (3)
C16—N2—C9—C8	170.31 (12)	C19—C20—C21—C16	0.5 (3)
C7—C8—C9—N2	−19.28 (13)	C17—C16—C21—C20	0.7 (2)
C7—C8—C9—C10	−142.80 (12)	N2—C16—C21—C20	−176.72 (15)

### Table 1 Y-X···Cg π ring interactions, Cg4 is the centroid of ring C16-C21, and Cg2 is the centroid of the ring C1-C6. [Symmetry codes: (i) 1-x,1-y,1-z ; (ii) -x,-1/2+y,1/2-z]

**Table:**

*X*–*H*···*Cg*X (Å)	X···*Cg,* (Å)	H···*Cg*	X···Perp (Å)
C9–H9···Cg4^i^	3.6677 (16)	2.82	2.76
C12–H12···Cg2^2^	3.6061 (18)	2.88	-2.79
